# Case report and narrative review of the literature: a rare colonic stent failure in a palliative patient

**DOI:** 10.3389/fgstr.2023.1279085

**Published:** 2023-10-11

**Authors:** Morgan Bressington, Alexander O’Connor, Karen Telford

**Affiliations:** ^1^ Department of General Surgery, Manchester University NHS Foundation Trust, Manchester, United Kingdom; ^2^ Faculty of Biology, Medicine and Health, The University of Manchester, Manchester, United Kingdom

**Keywords:** gastroenterology, palliative care, colonic stent, malignant large bowel obstruction, survival

## Abstract

**Introduction:**

With palliative patients, a holistic approach is important. Interventions should minimise length of hospital stay, maximise quality of life, and control symptoms. A self-expanding metal stent (SEMS) for the palliative treatment of malignant large bowel obstruction (MLBO) is designed to provide these benefits to patients approaching the end of their life. We present the case of a patient treated with a SEMS over 2 years earlier for MLBO. He was treated with palliative intent at diagnosis because his frailty and medical co-morbidities precluded surgery. He later presented with severe tenesmus, and these new symptoms were later found to be due to a rare stent failure in which the stent had fractured and was irretrievable. This had to be managed conservatively before the patient sadly passed away 7 months later.

**Discussion:**

A SEMS is considered the first-line treatment to relieve MLBO caused by inoperable left-sided colonic cancer. This treatment offers a reduced length of hospital stay, reduced stoma rates, fewer complications, and comparable survival compared to de-functioning stoma. However, SEMSs are not expected to be in use for extended periods of time. The literature reports an average survival after a colonic stent insertion of between 121 and 199 days when used in a palliative setting.

**Conclusion:**

This is one of the first case reports to describe a colonic stent failure occurring over 2 years after insertion. This case argues that further research into the longer-term outcomes of this management option is warranted, particularly as palliative patients are living longer.

## 1 Introduction

Bowel cancer is the fourth most common cancer in the United Kingdom and accounts for 11% of all new cancer cases (1). Globally, the annual incidence is estimated at 2 million, and it accounts for approximately 1 million deaths per year (2). With an ageing population, the care of palliative colorectal cancer patients continues to evolve whilst interventions are focused on improving quality of life (QoL). Surgeons are now treating more of these patients than ever before, with an increase in the non-operative management of colorectal cancer (3) and the development of surgical expertise and research in this field (4, 5).

Colonic self-expanding metal stents (SEMSs) are often used to treat malignant large bowel obstruction (MLBO) in patients presenting as an emergency who are not candidates for surgical intervention. A stent relieves the immediate risk of bowel obstruction and is commonly inserted under endoscopic and fluoroscopic guidance, with a success rate in excess of 90% (6). Relieving the obstruction with a SEMS avoids the risks associated with emergency surgery and stoma formation in patients who are often frail and present with medical co-morbidities (7).

Whilst the benefits of this procedure are apparent, it is important to understand the potential complications, particularly in patients who may live for longer than 12 months, when the evidence on the efficacy and complication rates are limited (7). Here we present a case of a rare late stent failure in line with the 2018 surgical case report (SCARE) guidelines (8).

## 2 Case presentation

For the purposes of this report, the patient will be referred to as “Michael”.

Michael was a 73-year-old man with severe chronic obstructive pulmonary disease (COPD), for which he required home oxygen therapy (4 L/min) and long-term azithromycin for recurrent infective exacerbations. He had an exercise tolerance of 10 yards limited by breathlessness, and he lived with his family, who were his carers. His clinical frailty score was 7 and his Eastern Clinical Oncology Group (ECOG) performance score was 3.

He was diagnosed as an emergency with an obstructing sigmoid colon cancer staged at T3 N1 M0 on computed tomography (CT). His co-morbidities precluded any surgical intervention at diagnosis and therefore he was treated with palliative intent. A flexible sigmoidoscopy was performed 2 days later using an Olympus™ gastroscope under sedation with 50 μg of intravenous fentanyl, which identified a malignant stricturing lesion in the distal sigmoid colon. Diagnostic biopsies were taken and an uncovered SEMS (Hanarostent® DNZL-20-140-230) was successfully placed under fluoroscopic and endoscopic guidance. Biopsies later confirmed moderately differentiated invasive adenocarcinoma. Following discussions at the colorectal cancer multidisciplinary team meeting it was decided that Michael was not a candidate for surgery or chemotherapy. A repeat CT scan 4 months later did not show any disease progression and his stent was patent and Michael was offered follow-up every 6 months.

He later presented as an emergency 30 months after diagnosis, describing reduced bowel frequency with darker and looser stool and severe tenesmus, neither of which were associated with any pain, nausea, or fresh rectal bleeding. Michael was an ex-smoker and a non-drinker and lived with his son and granddaughter. He now mobilised with a stick and had an exercise tolerance of 2 yards limited by shortness of breath. On examination, Michael had a soft abdomen with only mild lower abdominal tenderness. A digital rectal examination revealed a palpable colonic stent in the lower rectum.

**Figure 1:**
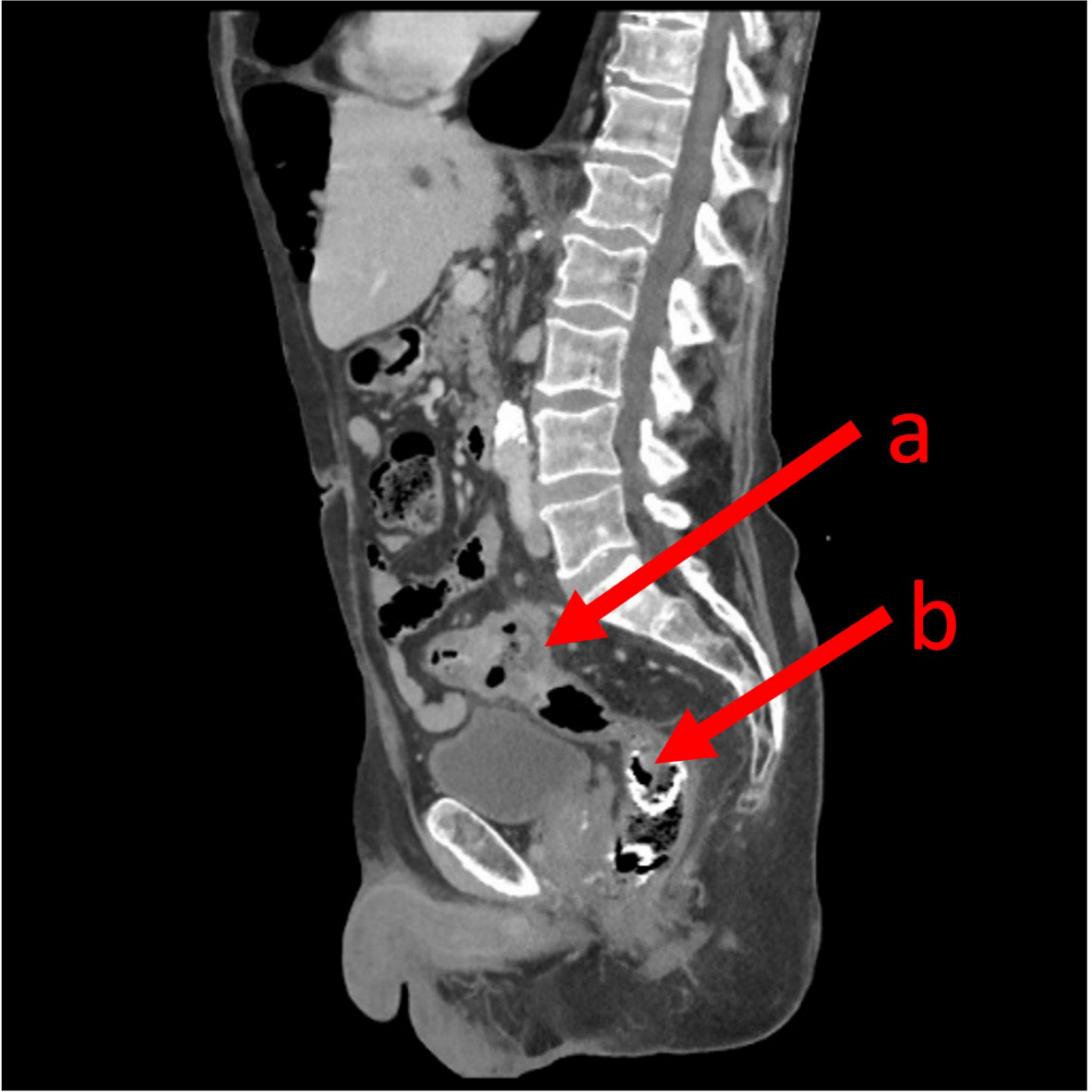
A sagittal computed topography image demonstrating the primary sigmoid colon cancer **(A)** and the SEMS in the lower rectum **(B)**.

**Figure 2:**
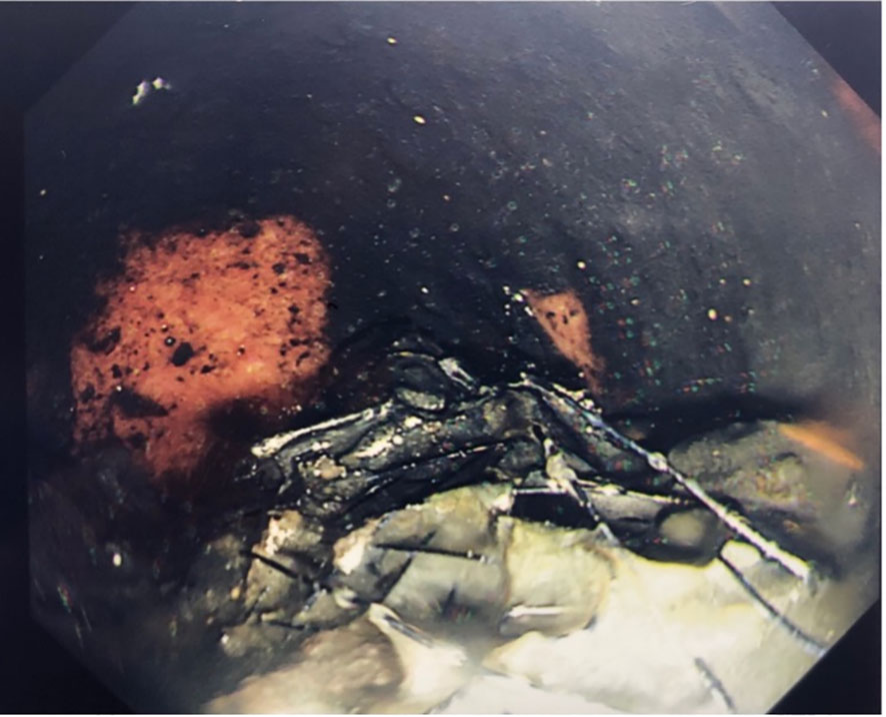
An endoscopic image demonstrating the stent displaced and snapped in the rectum with evidence of melaena.

A CT scan of the abdomen and pelvis was performed and demonstrated migration and a fracture of the colonic stent into the rectum without evidence of bowel obstruction (shown in **Figure 1**). The CT also identified progression of his colorectal cancer with new liver metastases. Further assessment of the stent was conducted by flexible sigmoidoscopy (Olympus™ Video Colonoscope CF-HQ290L) under light sedation with 2 mg of intravenous midazolam. The stent was found fractured and partly disintegrated, with part hanging into the rectum, and there was evidence of melaena (shown in **Figure 2**). The proximal aspect of the stent was adherent to the carcinoma, with tumour ingrowth making retrieval impossible without the risk of traumatising the sigmoid tumour.

The ongoing management of the stent failure was considered by the multidisciplinary team. Colonic SEMSs placed with palliative intent are not designed to be removed and it was felt that doing so risked catastrophic perforation or bleeding. It was considered that the stent may pass spontaneously during defecation given that it had already partially disintegrated, with a part hanging into the rectum. In addition, it was considered that given the failure of this stent, it may become necessary to place a further SEMS if obstruction were to recur.

Sadly, Michael’s health, and with it his fitness for any intervention, had deteriorated since his initial diagnosis. Even the most minimal of exertion was limited by severe breathlessness. Inpatient medical and social input was sought, and Michael’s respiratory function was optimised, his polypharmacy was rationalised, and further social support was put in place.

Following discussions with Michael and his family regarding the management of the stent in the context of disease progression and worsening physical fitness the decision was taken to “watch and wait”. Given that the stent was not causing an obstruction, a conservative management approach was initially deemed most appropriate. Michael was prescribed laxatives to avoid constipation and was discharged home. Michael was reviewed by telephone 7 months later and was not displaying symptoms of colonic obstruction and reported having passed a metallic object consistent with a stent 3 months earlier. He sadly passed away 1 month later.

## 3 Discussion

Approximately 10%–20% of patients with colorectal cancer present as an emergency with MLBO (9). Bowel resection is the only definitive management for patients treated with curative intent (10). In patients who are not immediately fit for surgery a SEMS can act as a bridge to surgery whilst the patient is medically optimised and appropriately counselled. For those treated with palliative intent, however, a SEMS offers a non-surgical management option to avoid stoma formation and is now the treatment of choice (6, 7).

A SEMS is considered the first-line treatment to relieve MLBO caused by left-sided colonic cancer that is either inoperable or identified in patients who are unfit for surgery—as in this case (11, 12). This treatment offers a reduced length of hospital stay, reduced stoma formation rates, fewer complications, and comparable survival compared with patients treated surgically with a stoma formation (7, 13, 14). This makes it an attractive treatment choice for palliative patients with no option of curative surgery. When compared with surgical decompression, SEMSs have demonstrated an improved QoL in the short and long term at 2 weeks and 12 months, respectively, in a series of 52 patients randomised between SEMS or surgical decompression (15). In a similar series of 30 patients randomised between SEMS and stoma formation, the SEMS group demonstrated better QoL than the stoma group and, overall, the healthcare cost was 6.9% lower (16). However, SEMSs are not without potential complications, with the risk of perforation reported to be approximately 7.4% (17), and higher in patients receiving chemotherapy (18). At the 12-month follow-up, van den Berg et al. reported SEMS-related mortality as high as 38% in a series of 48 patients, with all cases related to SEMS perforation (19).

There is also evidence to support the use of SEMSs as a bridge to surgery in patients with MLBO who are deemed potentially curable at diagnosis (7). These patients, treated with a stent, instead of temporary stoma formation, have a shorter length of hospital stay following the decompressive procedure and later, after definitive colonic resection (20).

Colonic stents are not expected to be in use for extended periods of time. The literature reports an average survival after a colonic stent insertion of between 121 and 199 days when used in a palliative setting (21). Fiori et al., in a unique series of 22 patients randomised between SEMS or stoma formation for unresectable colorectal cancer, continued follow-up until death, and reported a median survival of 280 days (range 135–591 days) and no difference between the two groups (12). Stents used as a bridge to surgery remained in place for between 1 and 4 weeks prior to definitive surgery (22, 23). Therefore, few data exist on the long-term efficacy and durability of SEMSs, with an average follow-up period of 14 months after stent placement for “long-term” studies (13). The largest reported randomised controlled trial regarding the use of colonic stents in MLBO, the Colorectal Endoscopic Stenting (CreST) trial, used a follow-up period of 12 months (7). The same follow-up period is used in the CreST2 trial, currently ongoing, examining the use of covered or uncovered SEMSs in a palliative setting (24). Our patient re-presented to the emergency department after 30 months, a follow-up period rarely observed in the literature.

Studies exploring the long-term outcomes of SEMSs have identified a 9% late complication rate (defined as >30 days): these complications included stent migration, blockages, and colo-vesical fistulation (25). In a recent multicentre trial, stent failure was found to occur, on average, at 19 weeks following insertion (23). The study in question had an average follow-up of 26 weeks and a maximum follow-up of 80 weeks. The stent failure described in our case falls well outside this time frame. Whilst local guidelines exist concerning the follow-up of patients treated with curative intent, no such guidelines exist for the follow-up of palliative patients; however, in our unit, Michael and other patients like him are routinely offered a review every 6 months.

This is one of the first case reports to describe a colonic stent failure occurring over 2 years after insertion. It also highlights that there exists a cohort of palliative patients with malignant colonic cancers treated with SEMSs who go on to live longer than the maximum follow-up period reported in the literature. Given the paucity of the literature concerning colonic stents in place for more than 12 months, little is known about the potential future complications, an example of which is reported here. This case argues that further research into the longer-term outcomes of this management option is warranted, particularly as palliative patients are living longer: one large South Korean series of 400 patients with left-sided MLBO treated with SEMSs reported a median overall survival of 18.23 months (95% confidence interval: 14.61–21.85 months) (26).

The CReST2 trial is actively recruiting and will eventually provide a more robust evidence base on the use of either covered or uncovered SEMS for MLBO treated with palliative intent. However, as discussed earlier, the follow-up period in this trial is only 12 months and may not capture these rare late complications in patients who survive longer.

## 4 Conclusion

This case highlights a potential late complication of an uncovered colonic stent placed with palliative intent in a patient with MLBO. Given that these palliative patients are living for longer, we advocate further research into the outcomes of this cohort who go on to live for more than 12 months, which are rarely reported in the literature.

## Data availability statement

The original contributions presented in the study are included in the article/supplementary material. Further inquiries can be directed to the corresponding author.

## Ethics statement

Written informed consent was obtained from the individual(s) for the publication of any potentially identifiable images or data included in this article.

## Author contributions

AO’C: Methodology, Supervision, Writing – review & editing. MB: Conceptualization, Data curation, Writing – original draft. KT: Supervision, Writing – review & editing.
